# Magnetic resonance probing of ground state in the mixed valence correlated topological insulator SmB_6_

**DOI:** 10.1038/s41598-018-25464-y

**Published:** 2018-05-08

**Authors:** S. V. Demishev, M. I. Gilmanov, A. N. Samarin, A. V. Semeno, N. E. Sluchanko, N. A. Samarin, A. V. Bogach, N. Yu. Shitsevalova, V. B. Filipov, M. S. Karasev, V. V. Glushkov

**Affiliations:** 1 Moscow Institute of Physics and Technology, Dolgoprudny, 141700 Moscow region, Russia; 2 Prokhorov General Physics Institute of the Russian Academy of Sciences, Moscow, 119991 Russia; 3 National Research University Higher School of Economics, Moscow, 101000 Russia; 4 Institute for Problems of Materials Science of NASU, Kiev, 03680 Ukraine

## Abstract

Introducing of topological insulator concept for fluctuating valence compound – samarium hexaboride – has recently initiated a new round of studies aimed to clarify the nature of the ground state in this extraordinary system with strong electron correlations. Here we discuss the data of magnetic resonance in the pristine single crystals of SmB_6_ measured in 60 GHz cavity experiments at temperatures 1.8–300 K. The microwave study as well as the DC resistivity and Hall effect measurements performed for the different states of SmB_6_ [110] surface prove definitely the existence of the layer with metallic conductivity increasing under lowering temperature below 5 K. Four lines with the g-factors g ≈ 2 are found to contribute to the ESR-like absorption spectrum that may be attributed to intrinsic paramagnetic centers on the sample’s surface, which are robust with respect to the surface treatment. The temperature dependence of integrated intensity *I*(*T*) for main paramagnetic signal is found to demonstrate anomalous critical behavior *I*(*T*) ~ (T^*^ − *T*)^ν^ with characteristic temperature T^*^ = 5.34 ± 0.05 K and exponent ν = 0.38 ± 0.03 indicating possible magnetic transition at the SmB_6_ [110] surface. Additional resonant magnetoabsorption line, which may be associated with either donor-like defects or cyclotron resonance mode corresponding to the mass *m*_*c*_ ~ 1.2*m*_0_, is reported.

## Introduction

In spite of long-lasting history of studies, the mixed valence compound samarium hexaboride, SmB_6_, remains a puzzle maker^1^. The mixed valence state of SmB_6_ is characterised by rapid charge fluctuations between Sm^3+^ and Sm^2+^ states with the temperature dependent Sm^3+^/Sm^2+^ ratio, which is slightly greater than 1:1^1^. Indirect hybridization gap ~20 meV opening below 40 K and intragap donor states placing ~3 meV below the conduction band were reliably established from numerous transport and optical experiments (see^2–6^ and references therein). Some exciting low temperature properties, which include (but are not limited to) a famous resistivity saturation^1^ and amazing zeroing of Seebeck coefficient at *T* < 5 K^6,7^, have stimulated development of several competing theoretical approaches. Initial suggestion for low temperature Wigner crystallization^8^ was followed by Kondo insulator (KI) model^9,10^, which became the most popular explanation of the rich physics occurring in SmB_6_. The KI model treats the intragap states as the ones induced by some impurities. However, the direct measurements of complex optical conductivity^4,5^ showed clearly that the spectral features corresponding to the 3 meV intragap states disappear completely at temperatures above ~16 K. This behavior can be hardly explained by any persistent impurities or defects. At the same time, the temperature evolution of the intragap states can be well understood within exciton-polaron (EP) model^11^, which was initially developed to explain the origin of extra inelastic peaks in the magnetic neutron scattering spectra of SmB_6_^12^. Within the EP model these intragap states correspond to the bound excitations between the 5d electron and hole in the 4f shell of Sm^3+^ dressed by polaronic “cloud” while saturation of resistivity results from the transition into a coherent state of these strongly interacting exciton-polaron complexes^5^. On the contrary, the KI model does not provide any consistent explanation of the aforementioned “5 K anomaly”, at which a whole number of features in thermal expansion coefficient, NMR spin-lattice relaxation time, elastic modulus and magnetoresistance was reported in addition to resistivity plateau and thermopower zeroing (see ref.^5^ and references therein).

At that time the mainstream of the SmB_6_ studies was to treat the low temperature properties as corresponding to the sample bulk^2–6,8,10,11^, and the experimental fact that the low temperature properties depend on the SmB_6_ surface treatment^13^ was ignored *per se* in any theoretical interpretation. A real breakthrough in understanding of SmB_6_ physics has recently occurred with introducing of the topological Kondo insulator (TKI) concept^14,15^. It has been realized that a non trivial topological *Z*_2_ invariant may appear for the band structure of SmB_6_ if the average valence of Sm exceeds some critical value of 2.56^15^. Allowing for the relative content of the Sm^3+^ and Sm^2+^ ions this material was suggested to be a very probable TKI candidate. Within this approach the sample bulk is a Kondo insulator with a gap ~20 meV while the itinerant electrons may be attributed to the states of the metallic sample surface. This emerging model explains naturally the low temperature resistivity saturation and thermopower damping. The surface origin of the resistivity plateau was unambiguously confirmed in the charge transport studies of the SmB_6_ samples of various thicknesses^16,17^. The advantage of TKI approach over other theoretical models was also demonstrated by point-contact spectroscopy data^18^, where only TKI model allowed the precise quantitative simulation of the conductance spectra detected for the Ag-SmB_6_ junction. The band calculations^19^ confirmed the existence of topologically protected states on SmB_6_ surface with multiple Dirac cones located at the Γ and X points of the surface Brillouin zone. In agreement with theoretical calculations, the ARPES investigations^20,21^ revealed surface states with linear spectrum being a fingerprint of topological insulator (TI).

Nevertheless, in spite of successful application of the TKI model for the case of SmB_6_, it was recently argued that this material is a trivial surface conductor rather than topological one^22^. So the present state of the art of SmB_6_ studies leaves several points requiring further clarification. First of all, the metallic layer at the sample surface is currently understood as possessing a temperature independent resistance *R*_*s*_(*T*) = const^18^. Any “standard metallicity” (decreasing of *R*_*s*_(*T*) with temperature lowering) below 5 K has, to our best knowledge, never been reported. In this respect, some alternative explanations of the surface conductivity of SmB_6_ like polarity-driven surface metallicity^23^ or exotic hopping transport with zero activation energy^24^ may be considered as well. Moreover, the reported evidence for the Fermi surface in SmB_6_ proved by quantum oscillations experiments^25,26^ was recently put into question^27^. Simultaneously the linear *T* contribution to the specific heat of SmB_6_ cannot be attributed to the surface states^28,29^.

Secondly, the problem of “5 K anomaly”, which was well established in the earlier studies^5,6,8^, attracts little attention in the modern TKI concept. This temperature region is usually treated as an accidental crossover caused by the interplay between surface and bulk conductivity rather than as any real transition in this strongly correlated system^17^.

The last but not the least important issue is related to the electron spin resonance (ESR) in the pristine SmB_6_ that was observed for the first time at liquid helium temperatures in 1996^30^ and was neglected by all the theoreticians to date. Due to valence fluctuations the bulk matrix of SmB_6_ was believed to be ESR silent, and ESR signal was observed only in samples doped specially with S-state magnetic ions like Eu^2+^ or Gd^3+^ (see^30^ and references therein). In ref.^30^, a complicated ESR spectrum consisting of several lines was reported for pure SmB_6_ in the frequency range 40–120 GHz. This phenomenon was interpreted as due to some intrinsic paramagnetic defects, which exist in the whole bulk of the sample. Unfortunately, these experiments were carried out at two fixed temperatures (1.8 K and 4.2 K) only^30^ so they did not allow extracting any contribution of the surface states to the observed resonant magnetoabsorption spectra.

Nevertheless, the presence of paramagnetic defects or impurities on the surface is very crucial for TI physics. These magnetic centers break time-reversal symmetry (TRS) removing the topological protection of the surface states (SS). As a result, the initial 2D massless Dirac spectrum should evolve to a gapped one so that the quasiparticles in the surface layer acquire a finite effective mass^31–35^. In addition, magnetic impurities at the TI surface may order ferromagnetically^33–35^ although some recent experiments^36^ do not confirm this theoretical expectation and the band gap at Dirac point for some magnetic topological insulators may have non-magnetic origin. In this respect, the study of the SS evolution under doping by magnetic impurities in SmB_6_ may be considered as an additional check for topological protection of the SS in this material^16^. Moreover, taking into account the results of ref.^30^, it is possible to suppose that the mixed valence TKI SmB_6_ possesses some “built-in” mechanism of the TRS breaking due to the presence of intrinsic paramagnetic centers. As a result, a pure Dirac spectrum *E* = *v*_*F*_ · *p* linear in momentum should change to the dispersion law for the energy $E=\sqrt{{({v}_{F}p)}^{2}+{{\rm{\Delta }}}^{2}}$ ^35^, with the energy gap 2Δ and Fermi velocity *v*_*F*_, producing drastic changes in macroscopic surface conductivity.

**Figure 1:**
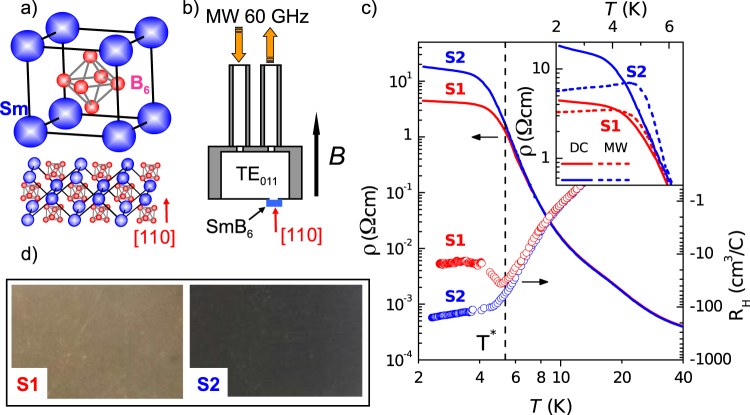
Unit cell and [110] surface of SmB_6_ (**a**), experimental layout used for microwave experiments (**b**); DC resistivity and Hall effect data (**c**) and the views of the SmB_6_ [110] surface states S1 and S2 in optical microscope with 4x magnification (**d**). Inset in panel c presents the comparison of the DC resistivity and inversed microwave conductivity for the different states of [110] surface.

In the present work we are aimed to address the aforementioned problems by studying the magnetic resonance. Although some ESR experiments were performed for the “classic” TIs^37^ there are no modern studies of the ESR in the strongly correlated limit of fluctuating valence at present. Here the temperature evolution of magnetic resonance measured for pristine single crystals of SmB_6_ in 60 GHz cavity experiments is reported. Following to the ideas of ref.^10^ we examined two different states of the SmB_6_ [110] surface (Fig. 1a), which are denoted as S1 and S2 (Fig. 1d), to elucidate any related surface effects. These states were obtained by different combinations of sample polishing and chemical etching (see Methods section). The difference between polished mirror-like S1 and etched S2 states is clearly visible by eye as showed in sample surface pictures corresponding to 4 times magnification (Fig. 1d).

The effect of different surface preparation can be clearly recognized in DC transport properties (Fig. 1c). The S1 → S2 evolution results in the fourfold enhancement of DC resistivity at *T* ~ 2 K and the difference between temperature dependences ρ(*T*) for S1 and S2 states develops below 6 K (Fig. 1c). It is worth noting that the ρ(*T*) curves in the both cases keep the typical shape for SmB_6_, which is usually explained in parallel resistors model, 1/*R*(*T*) = 1/*R*_*s*_ + 1/*R*_*b*_(*T*) (*R*(*T*) and *R*_*b*_(*T*)) denote total sample resistance and bulk contribution to the resistance, respectively)^17^. Thus, resistivity data suggest that surface conductivity in the S2 state is lower than in the S1 state.

Hall effect is also sensitive to the surface treatment. Although any used surface preparation keeps the negative sign of Hall coefficient *R*_H_, its absolute value for *T* ~ 2 K in the S2 state increases by an order of magnitude with respect to the S1 state (Fig. 1c). Similar to resistivity, the most difference in the temperature dependences *R*_H_(*T*) for the S1 and S2 states develops in the plateau region (Fig. 1c). However, the decomposition of the integral Hall coefficient into bulk and surface parts is not straightforward and depends on the way by which sample bulk and surface layer contribute to the total Hall voltage. Therefore, in the present work, Hall coefficient is considered as a qualitative indicator, which may suggest that electron concentration at the S2 surface is less than at the S1 surface.

Our microwave cavity measurements were performed with the help of experimental layout, where the bottom of reflecting cylindrical cavity operating at TE_011_ mode is made of thin metallic copper or silver foil with a small hole. The hole located at the maximum of the oscillating magnetic field is closed from the outside by the sample fixed at the cavity bottom by conducting silver paint (Fig. 1b). As long as only the central part of the sample is exposed to a microwave field inside the cavity, the considered layout excludes any inhomogeneity of a steady magnetic field *B* inside the sample that would result in a distortion of the ESR line shape^37–39^. Additionally, this experimental schema allows calculating the microwave (MW) conductivity for the studied sample σ_MW_(*T*) from the temperature dependence of the cavity losses in zero magnetic field^38–40^.

In the case of SmB_6_, it is possible to notice that the layout described allows checking the presence of surface metallic layer expected for TI. Indeed, in the framework of the parallel resistor model it is possible to separate bulk and surface conductivity at low temperatures and estimate the relative part of the MW power absorption inside the sample surface *P*_*s*_ and in the sample bulk *P*_*b*_ (see Supplementary Figure 1 and Supplementary Note 1). In our experimental conditions *P*_*s*_ ≈ *P*_*b*_ at *T* = 6 K, and the absorption at the surface turns out to be more than 3 times higher than in the sample bulk for *T* ~ 5 K, where the bulk and surface contributions to sample DC resistivity are almost the same (see Supplementary Materials). Temperature lowering at *T* < 5 K results in rapid freezing out of the bulk absorption. For example, only about 2% of the total microwave power is absorbed in the sample bulk at *T* ~ 4 K (see Supplementary Figure 1). Thus, our model calculation suggests strongly that the surface metallic layer should be predominantly coupled to a MW field in the plateau region, and cavity measurements allow to extract the contribution of the surface conductivity from experiments.

Data presented in the inset to the panel c in Fig. 1 for the surface [110] demonstrate clearly that ρ(*T*) *increases* and 1/σ_MW_(*T*) *decreases* with temperature for both surface states (S1 and S2) up to 4 K. In this temperature interval the surface absorption of MW radiation dominates and the bulk absorption is negligible. Therefore the discrepancy between ρ(*T*) and 1/σ_MW_(*T*) may be attributed to a real “metallic” temperature dependence of the surface conductivity, which is different from the common assumption *R*_*s*_ = const. Additionally, the ρ(*T*) and 1/σ_MW_(*T*) almost coincide in the bulk conductivity region *T* > 6 K (inset in Fig. 1c) due to prevailing absorption in the sample bulk. We wish to mark that the decrease of the surface resistivity with lowering temperature was neither observed before nor used in any existing models of the SmB_6_ surface^16,17^. At the same time the “metallic” behavior of the surface resistivity following from the present work is consistent with the TKI model^14,15^ predicting persistent metallic layer at the SmB_6_ surface.

**Figure 2:**
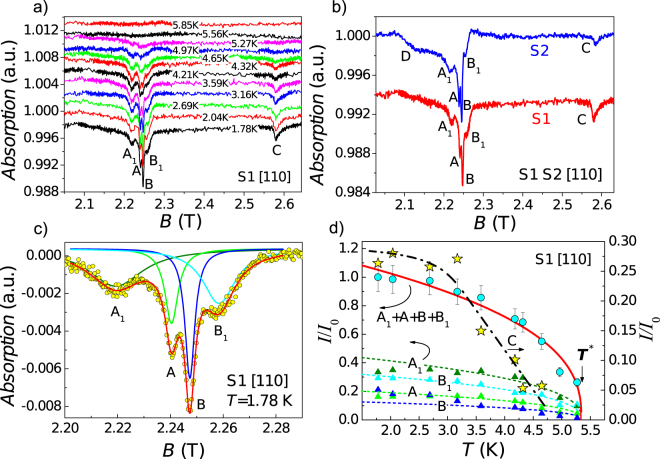
Temperature evolution of the resonant magnetoabsorption spectra for the S1 state of [110] surface of SmB_6_ (**a**); effect of surface treatment (**b**); line shape analysis for the main paramagnetic signal in the S1 state (**c**) and the temperature dependences of the integrated intensities for various spectral components (**d)**, see text for details.

When discussing the magnetic resonance data, let us first consider the case of [110] surface in S1 state (Fig. 2a). The observed spectra consist of several lines, which include the doublet A, B accompanied by satellites A_1_, B_1_ and extra line C. The amplitudes of these resonances increase with lowering of temperature, whereas the resonant fields remain constant within experimental accuracy. The position of the main doublet A, B agrees reasonably with the data for two main resonance lines reported previously in ref.^29^. It is worth noting that the observed spectra correspond to the excitation geometry of paramagnetic resonance, where oscillating electric field is missing and magnetic resonances are caused by applying of the oscillating magnetic field perpendicular to the steady one (Fig. 1b). Therefore the observed lines should have a paramagnetic nature either *per se* or at least in view of excitation method.

The effect of surface treatment on the observed spectra is illustrated by Fig. 2b. On a qualitative level, the lines A, B and A_1_, B_1_ remain almost unchanged. However, it is not possible to change the sample surface in the cavity experiment without dismounting and mounting the crystal again. Therefore, the coupling to the cavity in the measurements of the S1 and S2 states may be different even for the same crystal. For that reason, the influence of the SmB_6_ surface treatment on the amplitude of the main paramagnetic signal can be hardly established with any reasonable accuracy. Nevertheless, one can notice that the S1 → S2 transformation results in the twofold suppression of the relative amplitude of the line C with respect to the A-B doublet (Fig. 2b). In addition, the broad line D in the low field region appears for the S2 surface (Fig. 2b). Thus it is possible to conclude that the resonant magnetoabsorption spectrum undergoes both quantitative and qualitative changes due to the sample surface treatment.

We have carried out line shape analysis for the main paramagnetic signal in the S1 state. The sum of four lorentzians lines reproduces our experimental curves for A_1_, A, B, and B_1_ resonances very well and allows extracting the temperature dependences of line widths *W* and integrated intensities *I* (Fig. 2c). As long as the strongest temperature variation corresponds to the case of the first parameter, we shall concentrate on the analysis of the *I*(*T*) data only. The peculiarities of spin relaxation processes in SmB_6_, which determines the *W*(*T*) temperature evolution, will be published elsewhere.

The raw data for the integrated intensities are summarized in Fig. 2d. Interesting that all the lines are not observed at temperatures above 5.5 K. Moreover, the integrated intensity can be fitted rather well by a critical behavior

1 $$I(T) \sim {({T}^{\ast }-T)}^{v}$$

with characteristic temperature T^*^ = 5.34 ± 0.05 K and exponent ν = 0.38 ± 0.03. This model approximation is shown by the colored solid and dashed lines in Fig. 2d, where triangles correspond to *I*(*T*) dependences for the components A_1_, A, B, B_1_ and circles present the total integrated intensity for the whole paramagnetic signal A_1_ + A + B + B_1_. It is worth noting that the temperature *T* ^*^ correlates well with the onset of the surface conductivity and the saturation of Hall effect (Fig. 1c). The behavior of the *I*(*T*) dependence for the line C (stars in Fig. 2d) is different from that of the paramagnetic A–B signal. Lowering temperature results in a linear increase of the integrated intensity, which is followed by its saturation for *T* < 3 K. Simultaneously, this mode is proved to be sensitive to the sample surface state (Fig. 2b).

**Figure 3:**
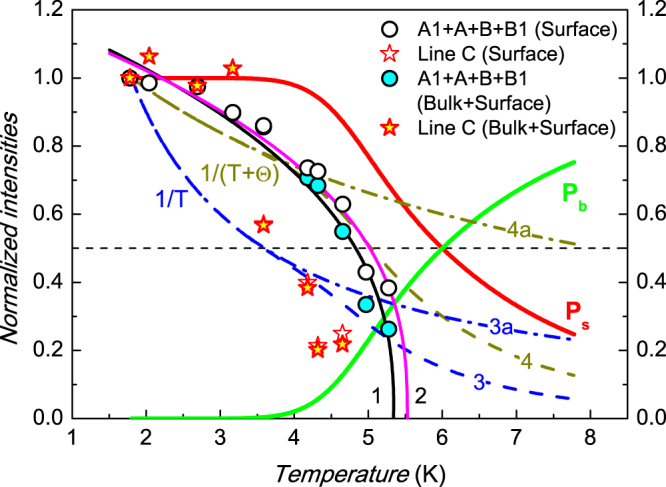
The comparison of the possible effects of MW radiation absorption at the SmB_6_ surface and in the sample bulk. Open symbols correspond to raw integrated intensities normalized to the value at *T* = 1.8 K; symbols filled by colors denote the recalculated values assuming dominating surface contribution. Curves 1, 2 are the best fits obtained with the help of Equation (1); curves 3, 4 are the temperature dependences expected for Curie and Curie-Weiss spin susceptibilities assuming dominating surface contribution. Curves 3a and 4a are the same as curves 3 and 4 for the case when paramagnetic centers are uniformly distributed in the sample (see text for details).

To elucidate the nature of different modes observed in our magnetic resonance experiments, it is instructive to consider the MW power absorption redistribution between bulk and surface states with lowering temperature (Fig. 3). The integrated intensities scale with absorbed MW power and, consequently, the observed integrated intensity can be expressed as a weighted sum *I*(*T*) = *P*_*b*_ · *I*_b_(*T*) + *P*_*s*_ · *I*_*s*_(*T*), where *I*_b_(*T*) and · *I*_*s*_(*T*) denotes integrated intensities due to absorption in the bulk and at the surface respectively, and *P*_*s*_ + *P*_*b*_ = 1. As long as *P*_*s*_ increases and *P*_*b*_ decreases upon temperature lowering (Fig. 3), the enhancement of the magnetic resonance signals at low temperatures means that the observed modes of magnetic oscillations may either be equally caused by both bulk and surface absorption (*I*(*T*) = *I*_*b*_(*T*) = *I*_*s*_(*T*)), or originate mainly from the sample surface (*I*(*T*) ≈ *P*_*s*_(*T*)*I*_*s*_(*T*)). In the first case, the *I*(*T*) dependence reproduces the real temperature dependence of corresponding modes, whereas in the second case the true curve *I*_*s*_(*T*) is modulated by *P*_*s*_(*T*).

In order to check the latter opportunity we have calculated the expected *I*_*s*_(*T*) dependence as *I*(*T*)/*P*_*s*_(*T*) and compared with our initial *I*(*T*) data for the main paramagnetic signal and line C. The result is presented in Fig. 3, where white and colored symbols correspond to *I*_*s*_(*T*) and *I*(*T*), respectively. It is visible that our suggestion about surface origin of magnetic resonances does not modify the temperature dependence of the integrated intensity significantly. For example, the initial fit of *I*(*T*) by Equation (1) (curve 1 in Fig. 3) applied to *I*_*s*_(*T*) gives the parameters T^*^ = 5.53 ± 0.03 K and ν = 0.37 ± 0.03 (curve 2 in Fig. 3). This apparently is due to the fact that in the MW experiment in the plateau region the response from the surface layer plays a major role.

Another important question is the possibility of an alternative description of the *I*(*T*) dependence for the main paramagnetic signal. Assuming that ESR corresponds to some paramagnetic defects at the surface, it is possible to expect that *I*_*s*_(*T*) ~ χ(*T*)*P*_*s*_(*T*), where χ(*T*) denotes spin susceptibility. The model approximation for Curie law χ(*T*) ~ 1/*T* (curve 3 in Fig. 3) clearly show that this assumption does not meet experimental results. The use of Curie-Weiss law χ(*T*) ~ 1/(*T* + θ) with θ = 4.5 K allows better approximation of the *I*_*s*_(*T*) for *T* < 4 K than χ(*T*) ~ 1/*T* (curve 4 in Fig. 3). However, in this case it is not possible to explain why the ESR signal is observed at *T* = 5 K and is not observed at *T* = 6 K, although expected *I*_*s*_(*T*) ~ χ(*T*)*P*_*s*_(*T*) dependence (curve 4 in Fig. 3) suggests comparable integrated intensity at these temperatures. In addition, we wish to mark that the case of paramagnetic centers, which belong equally to the surface and bulk states, when *I*(*T*) ~ χ(*T*) does not fit well our experimental data (see curves 3a and 4a in Fig. 3 corresponding to the cases χ(*T*) ~ 1/*T* and χ(*T*) ~ 1/(*T* + θ) respectively).

The above consideration strongly supports the conclusion that the critical behavior of the integrated ESR intensity results from the temperature dependence of the spin susceptibility of paramagnetic centers to be responsible for ESR in SmB_6_. This means that these paramagnetic centers do not exist in the range *T* > T^*^ and may emerge in the sample below T^*^ due to some abrupt structural/magnetic transition. In our experiments, this transition is observed at the [110] metallic surface of SmB_6_, although experimental data do not exclude that the same effect takes place in the sample bulk. In addition these paramagnetic centers do not only exist at the sample surface, but are also robust with respect to surface treatment. Therefore in view of topologically protected nature of SmB_6_ surface, they may have intrinsic origin rather than extrinsic. As long as T^*^ ~ 5 K, the results obtained in the present work demonstrate the importance of re-considering of “5 K anomaly” in context of the modern topological physics of SmB_6_.

Full interpretation of the ESR in SmB_6_ requires development of a new theory which is beyond the scope of the present study. Here we consider an opportunity when some magnetic Sm^3+^ ions act as a source of intrinsic localized magnetic moments (LMM) visible in ESR experiments. According to ref.^41^ the ground state of *J* = 5/2 Sm^3+^ splits in the crystal electric field into Γ_7_ doublet and lowest Γ_8_ quartet. The relevant ESR theory was considered by Schlottmann^42^ for the case of CeB_6_, which has the same crystal structure as SmB_6_ and Γ_8_ ground state of magnetic Ce^3+^ ion. In the absence of quadrupolar ordering effects specific to CeB_6_ and missing in SmB_6_, the Γ_8_ state should correspond to four ESR modes^42^. This prediction meets the experimental situation (Fig. 2), where ESR spectrum in SmB_6_ is formed by four lines with the g-factors g(A_1_) = 1.944 ± 0.001, g(A) = 1.926 ± 0.001, g(B) = 1.920 ± 0.001 and g(B_1_) = 1.911 ± 0.001. These values are somewhat lower than the theoretical values for Γ_8_^42^, but similar effect occurs in CeB_6_, where ESR experiment also suggest g-factor to be very close to 2^43^. Therefore, our hypothesis concerning “Sm^3+^ origin” of ESR-active LMM does not contradict to the known experimental and theoretical data. However we wish to emphasize that not each Sm^3+^ ion could contribute to observed ESR signal. From the static magnetization data at *T* = 2 K it is possible to expect the average bulk number ~0.06% of Sm^3+^ ions to be responsible for ESR, whereas this number may be enhanced by ~30% at the topologically protected surface of SmB_6_ (see Supplementary materials).

Now let us consider the possible nature of the C and D lines, which are sensitive to the surface preparation (Fig. 2). Broad line D is found in the S2 state exclusively and may appear due to specific defects with the g-factor g ~ 2.02 induced by chemical etching at the sample surface. The line C is observed in both surface states, but its amplitude is lower for the S2 state, which is expected to have lower electron concentration than the S1 state. Thus, the C line is also due to ESR on some donor-like defect states in the surface layer, which are responsible for electron doping.

However, the temperature dependence of the C line (Figs. 2 and 3) is different from that of the main paramagnetic signal and does not meet the case of either Curie or Curie-Weiss law. It is possible to notice that the integrated intensity saturates in the interval *T* < 3 K where *P*_*s*_ = 1 and bulk states does not contribute to this feature of resonant magnetoabsorption. Therefore it is not possible to exclude that this feature in the spectrum may originate from cyclotron resonance (CR) in the topologically protected surface rather than from ESR (Fig. 3). This intriguing opportunity is due to the fact that the surface of TI is characterized by strong coupling between spin and orbital degrees of freedom. In this situation, the theory of magnetic resonance in TI^44^ suggests that CR may occur via excitation of the paramagnetic system of the electron spins and, therefore, it may be observed in the experimental geometry corresponding to excitation of magnetic dipoles used in the present work (Fig. 1b). As long as position of the C line does not shift when the surface state (and presumably electron concentration) changes, the interpretation of this line by CR in the TI surface layer is in conflict with the assumption about the massless Dirac spectrum because in this case cyclotron mass must depend on energy and hence on the doping level. However, the real spectrum at the SmB_6_ surface in the interval *T* < T^*^ should be gapped due to presence of intrinsic paramagnetic defects. Therefore, when Fermi energy *E*_*F*_ is close to Δ in the gapped spectrum, the cyclotron mass is given by ${m}_{c}(E)={E}_{F}/{v}_{F}^{2} \sim {\rm{\Delta }}/{v}_{F}^{2}$ and therefore may not strongly depend on the electron concentration. Our experimental data for the line C (see Fig. 2a,b) correspond to the value *m*_*c*_ = 1.2*m*_0_, where *m*_0_ is a free electron mass. The uncertainty in *v*_*F*_ parameter determination for SmB_6_^45^ makes it difficult to estimate the possible gap parameter and to compare it with the characteristic temperature T^*^ unambiguously. Nevertheless, when taking Fermi energy for the SmB_6_ [110] surface *E*_*F*_ ~ 0.46 meV^7^ and assuming Δ ~ *E*_*F*_, the effective mass *m* = 1.2*m*_0_, expected from the results of the present work, should correspond to *v*_*F*_ ~ 2.6 · 10^3^ m/s. This value lies within interval 68 < *v*_*F*_ < 6 · 10^5^ m/s reported in the available literature^7^. In addition, *E*_*F*_/*k*_*B*_ ~ Δ/*k*_*B*_ ~ 5.3 K is pretty close to the transition temperature T^*^. This indicates that the observed onset of the paramagnetic signal may be correlated with the formation of the gapped energy spectrum at temperatures below “5 K anomaly”. In our opinion, extra work is required to confirm or rule out the suggested exotic explanation of the C line observed in the present work.

Summarizing up, microwave cavity experiments at frequency 60 GHz together with the DC resistivity and Hall effect measurements performed for different states of [110] surface of SmB_6_ revealed existence of the layer with metallic conductivity, which increases when temperature is lowered in diapason for *T* < 4 K. An ESR-like absorption spectrum consisting of four lines with the g-factors close to 2 is detected and may be attributed to intrinsic paramagnetic centers at the sample surface, which are robust with respect to the surface treatment. The integrated intensity temperature dependence for paramagnetic signal *I*(*T*) is found to demonstrate anomalous critical behavior, which indicates possible magnetic transition at the [110] surface of SmB_6_. This observation indicates that well known “5 K anomaly” may be induced by the abrupt change of the TI surface state rather than by any crossover phenomenon. Additional resonant magnetoabsorption line, which may be associated with either donor-like defects, or CR mode corresponding to the mass *m*_*c*_ ~ 1.2*m*_0_, is detected.

## Methods

The quality of the studied samples was controlled by X-ray and EPMA analysis. In order to elucidate surface effects, two different states hereafter denoted as S1 and S2 of the sample surface were examined. The state S1 was obtained by polishing of the sample surface with the help of diamond powders. During this process the powder grain size was gradually reduced from 7 microns to 1 micron and a mirror-like was obtained (see Fig. 1d). The state S2 have been prepared by chemical etching of the S1 surface in the 1:10 mixture of the nitric acid and distilled water. After such treatment the matted grey surface develops. The DC resistivity and Hall effect are measured by the four-probe technique at home-made installation described in^46^. Magnetic measurements up to 5 T have been carried out with the help of SQUID magnetometer MPMS-5 (Quantum Design).

For the microwave cavity measurements we have used experimental layout proposed in^37–39^ and schematically shown in Fig. 1b. In this geometry, the cavity bottom is made of thin copper foil with a small hole at the maximum of microwave magnetic field. The measured SmB_6_ crystal is mounted outside the cavity in a way to cover the hole. For good electrical contact the conductive silver paint is used to fix the sample to the foil. As long as only central part of the measured sample is accessible to the microwave field, the local field acting on spins inside the sample is almost homogeneous and can be easily corrected to demagnetization factor. For the spatially homogeneous sample, this layout allows absolute calibration of the ESR absorption and the subsequent finding of the whole set of spectroscopic parameters, including oscillating magnetization^37,39^. In the spatially inhomogeneous state, where surface and bulk states are different like in SmB_6_, this analysis is not possible. For that reason, in order to estimate the concentration of the paramagnetic centers we have implied static magnetization measurements as described in Supplementary materials. The σ_*MW*_ was calculated from the sample contribution to the cavity losses at the frequency ω in zero magnetic field, which gives the corresponding quality factor ${Q}_{Sample}^{-1}=A\cdot {(\omega /{\sigma }_{MW})}^{1/2}$. The details about determination of the calibration coefficient *A* can be found elsewhere^38,39^. In this procedure, the microwave conductivity σ_*MW*_ appears as an effective parameter depending of the both bulk and surface state conductivity and distribution of the microwave field inside the sample. Our estimates of absorption of microwave radiation at the sample surface and in the bulk (see Supplementary Materials) show that in our experimental conditions the main part of the microwave losses for *T* < 5 K is due to the metallic surface layer of TKI SmB_6_. In our magnetic resonance measurements, external magnetic field was aligned along [110] direction of the sample (see Fig. 1b). The cavity quality factor loaded with the sample for the TE_011_ mode was about 8 · 10^3^ for frequency ω/2π ≈ 60 GHz. The resonant magnetoabsorption was detected in the temperature range *T* < 6 K, where surface contribution to the sample conductivity is essential (see Fig. 2a).

## Electronic supplementary material

Supplementary materials
