# Supplementary material for: Magnetic resonance probing of ground state in the mixed valence correlated topological insulator SmB6
Source: Sci Rep. 2018 May 8;8:7125. doi: 10.1038/s41598-018-25464-y (PMC5940907; doi:10.1038/s41598-018-25464-y)
Supplement: Supplementary file 1 — Supplementary materials [file 41598_2018_25464_MOESM1_ESM.pdf]

Magnetic resonance probing of ground state in the mixed valence correlated topological insulator SmB_6

S. V. Demishev[1,3], M. I. Gilmanov[1,2], A. N. Samarin[2], A. V. Semeno[1,2], N. E. Sluchanko[1,2], N.A.Samarin[2], A.V.Bogach[2], N. Yu. Shitsevalova[4], V. B. Filipov[4], M.S.Karasev[2] and V. V. Glushkov[1,2]

[1] *Moscow Institute of Physics and Technology, Dolgoprudny 141700 Moscow region, Russia*

[2] *Prokhorov General Physics Institute of RAS, Moscow 119991, Russia*

[3] *National Research University Higher School of Economics, Moscow 101000, Russia*

[4] *Institute for Problems of Materials Science of NASU, Kiev 03680, Ukraine*

Supplementary materials.

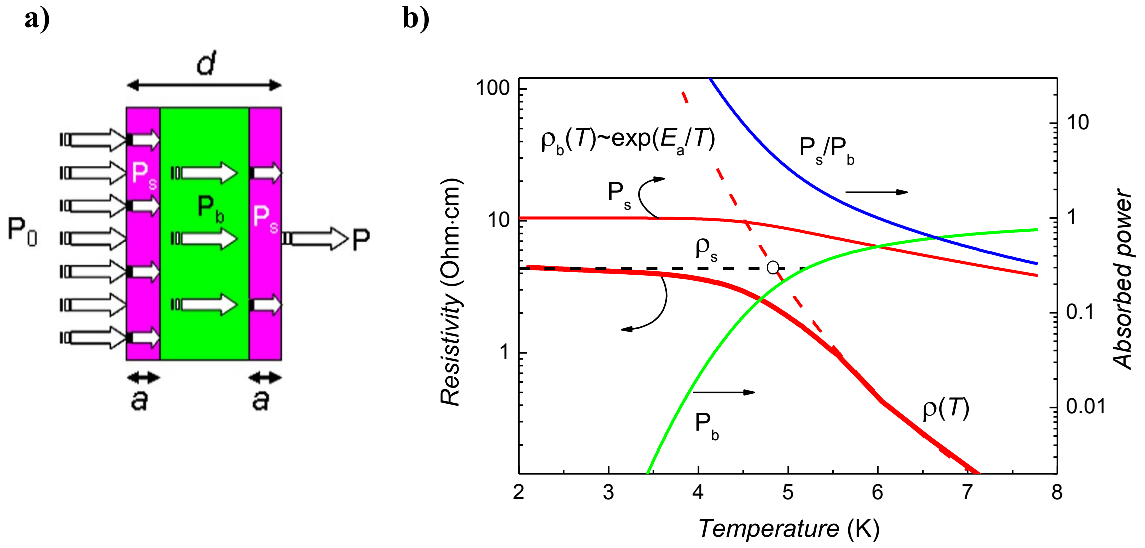

Supplementary Figure 1. Modeling of microwave power absorption in SmB_6 sample with finite thickness *d*. The incident electromagnetic wave is absorbed in the surface layers of the sample having thickness *a*, as well as in the sample bulk (panel a). In order to compute the relative parts P_s and P_b for the power absorbed at the surface and in the bulk respectively, it is necessary to separate bulk and surface conductivities contributing to DC resistivity temperature dependence $\rho(T)$. To estimate, we assume that bulk conductivity in the plateau region follows the law $\rho_b(T)$~$\exp(E_a/k_B T)$ denoted by red dashed line in the panel b. In this case the total conductivity from the surface layer $\rho_s(T)$=const (thus we neglect metallic temperature dependence of the surface layer shown in Figure 1 of the main text and possible deviations from the exponential law for the bulk states). The ρ_s value (black dashed line in panel b) is found by fitting of experimental data by equation $\rho(T)=1/[1/\rho_s+1/\rho_b(T)]$. This procedure gives $P_s(T)$, $P_b(T)$ and $P_s/P_b(T)$ temperature dependences shown in the panel b. It is visible that $P_s(T)$ and $P_b(T)$ are equal at T~6 K and lowering temperature results in the strong enhancement of the surface absorption with respect to the bulk. Namely, for T<4 K, almost all microwave power is absorbed inside the surface layer (see Supplementary Note 1 for calculation details).

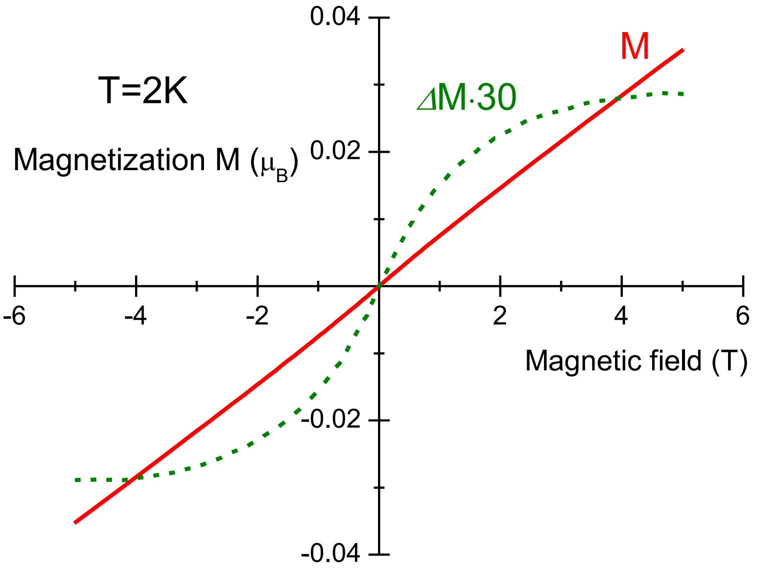

Supplementary Figure 2. Field dependence of magnetization M measured for the undoped SmB_6 single crystal with the surface state S1 at T_0=2 K (solid line). The dashed line presents the non-linear additive $\Delta M \equiv M_{sat}\varphi(B,T_0)=M(B,T_0)-aB$ with a= $6.75\cdot10^{-3}$ $\mu_B T^{-1}$. The magnetization data are given in Bohr magnetons per formulae unit (see Supplementary Table 1 and Supplementary Note 2 for details)

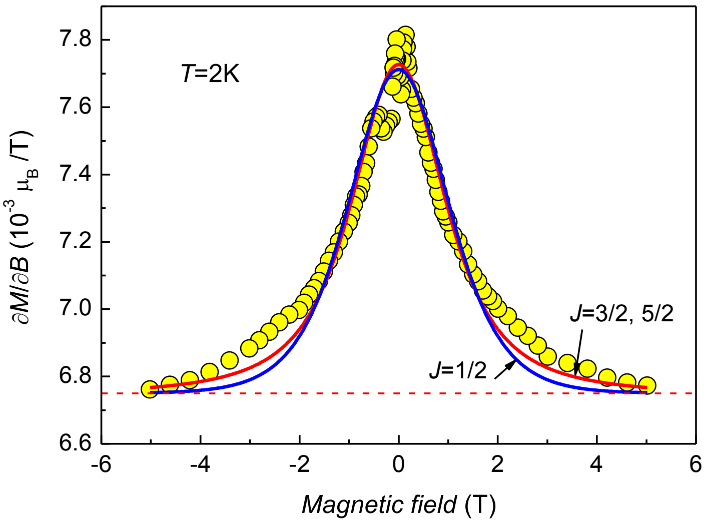

Supplementary Figure 3. The derivative $\partial M/\partial B$ at T=2 K. Points correspond to the experimental data; solid lines denote model fits for J=1/2, J=3/2 and J=5/2 obtained with the help of the derivative of Eq. 2s (fits for J=3/2 and J=5/2 are coinciding in the scale of the graph). The related parameters are listed in Supplementary Table 1. Dashed line corresponds to the value of parameter a in Eq. 2s (see Supplementary Notes 2,3 and 4).

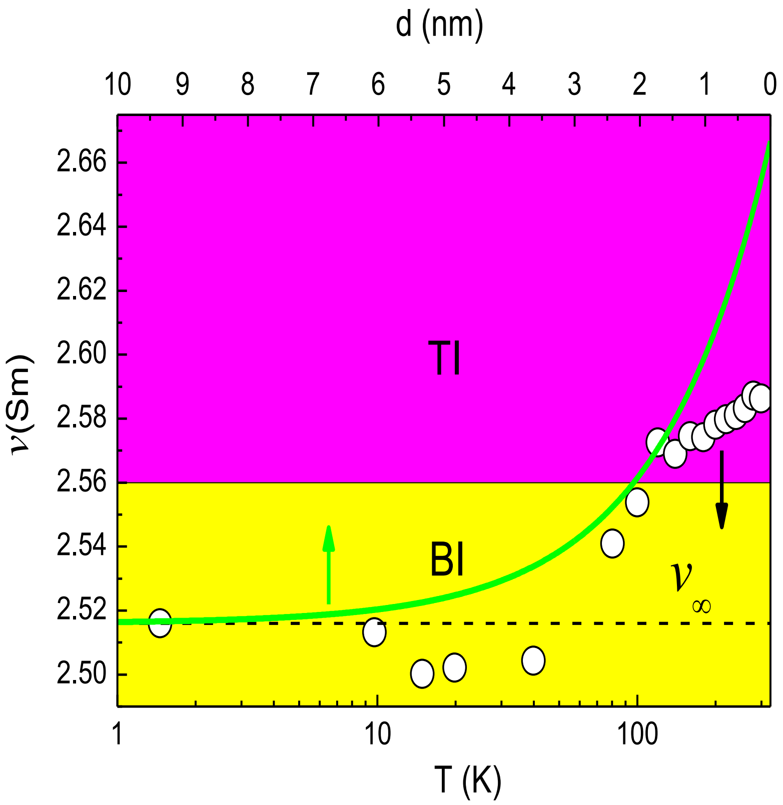

Supplementary Figure 4. Temperature dependence of Sm valence *v* in the sample bulk [Supplementary reference 1] (points) and calculated Sm valence *v*(*d*) as a function of the distance from the sample surface *d* profile [Supplementary reference 2] (solid line) assuming bulk value v_∞=2.516 [Supplementary reference 1]] (dashed line). Different colours on the graph denote the regions of the trivial bulk insulator (BI) and topological insulator (TI), drawn in accordance with the theory of cubic topological Kondo insulators (see Ref. 12 of the main text and Supplementary Note 5).

Supplementary Table 1.

	J=1/2	J=3/2	J=5/2
a (μ_BT^{-1})	(6.75±0.02)·10^{-3}	(6.75±0.02)·10^{-3}	(6.75±0.02)·10^{-3}
α (T^{-1})	0.75±0.03	1.25±0.03	1.57±0.07
M_{sat} (μ_B)	(1.27±0.05)·10^{-3}	1.68±0.03	(1.4±0.1)·10^{-3}
$N/N_{\rm Sm}$	(1.27±0.05)·10^{-3}	(5.6±0.1)·10^{-4}	(2.8±0.2)·10^{-4}

Parameters of the model calculation for T=2 K (see Supplementary Notes 3,4 and 5).

Supplementary Note 1: Model of the microwave power absorption in SmB_6 thin plate.

In the calculation, we assume that surface layer depth, a, is small with respect to the sample thickness, d. Indeed, in the topological Kondo insulator model, the parameter a is about size of the unit cell. As long as $d>>a$, it is possible to consider SmB_6 sample as δ-layer with the conductivity σ_s at the surface covering volume with the conductivity σ_b. For experimental layout used in cavity experiments (Figure 1 of the main text) it is sufficient to consider geometry shown in Supplementary Figure 1, panel a (i.e. the problem becomes essentially one-dimensional). In the case of semi-infinite sample ($d\to\infty$) the ratio of the microwave power absorbed in the front δ-layer, P_s, to the microwave power absorbed in the sample bulk, P_b, may be derived from a straightforward calculation, which yields $P_s/P_b=\sigma_s a/\sigma_b\delta_b$, where δ_b is the skin depth in the sample volume. The bulk conductivity $\sigma_b(T)=1/\rho_b(T)$ can be directly obtained from experimental data, whereas determination of σ_s from ρ_s is not so simple (see Supplementary Figure 1, panel b). In the parallel resistor model used for modeling of the $\rho(T)$ temperature dependence, the σ_s must be enhanced with respect to $1/\rho_s$ by the factor l_{eff}/a, where macroscopic length l_{eff} is comparable with the sample sizes and depends of the measurement method. For the standard four-probe schema applied for the sample having the shape of a rectangular parallelepiped, the effective length is the ratio of the sample cross-section square and cross-section perimeter. For the Van der Paw method l_{eff} is about $L/4$, where L is the distance between contacts at the sample surface. In any case, the final result acquires the form $P_s/P_b=\rho_b l_{eff}/\rho_s\delta_b$, and the ratio P_s/P_b does not depend on the thickness of the surface layer if the parameters ρ_b and ρ_s, following from experimental resistivity data, ρ_b and ρ_s, are introduced and $a<<l_{eff}$, δ_b. The finite sample thickness reduces the magnitude of the power absorbed in the sample bulk by $1-\exp(-2d/\delta_b)$. At the same time, in the considered case, the surface at the end of the sample will contribute to surface absorption as well (see Supplementary Figure 1, panel a), which will enhance P_s by $1+\exp(-2d/\delta_b)$. This gives

$$\frac{P_s}{P_b}=\frac{l_{eff}}{\delta_b\tanh(d/\delta_b)}. \qquad (1s)$$

For an estimate we have used expression $\delta_b=c\sqrt{\rho_b/2\pi\omega}$, which, in the plateau region can be re-written in the form $\delta_b=\delta_0\cdot f(T)$, where $\delta_0=c\sqrt{\rho_s/2\pi\omega}$ and $f(T)=\exp[E_a(1/T-1/T_0)/2k_b]$. The temperature T_0 denotes the crossing point of asymptotics ρ_s=const and $\rho_b\sim\exp(E_a/k_BT)$ (See Supplementary Figure 1, panel a). Thus Equation (1s) depends on two dimensionless parameters l_{eff}/δ_0 and d/δ_0 and resistivity activation energy E_a. Our experiments for the surface S1 correspond to the case $E_a\approx5$ meV, $l_{eff}/\delta_0=2.18$ and $d/\delta_0=1.25$, which gives temperature dependences $P_s(T)$, $P_b(T)$ and $P_s/P_b(T)$ shown in Supplementary Figure 1, panel a.

Supplementary Note 2: Low temperature magnetization field dependence.

The estimate of the paramagnetic centers concentration in the pristine SmB_6 with the S1 surface state may be obtained by the field dependence of magnetization $M(B)$ analysis. The $M(B)$ curve in SmB_6 is almost linear (Supplementary Fig. 2). Hereafter the magnetization is presented in the units of Bohr magneton per formulae unit. A small nonlinear contribution to $M(B)$, which becomes more pronounced at low temperatures (Supplementary Fig. 2), may be clearly detected in the data. For elucidating this part of $M(B)$, the derivative $\partial M/\partial B$ may be considered

(Supplementary Fig. 3). The data in Supplementary Figs. 2,3 indicate that magnetization of SmB_6 may be expressed by a superposition of two terms

$$M(B) = M_{sat}\varphi(B,T) + a \cdot B, \qquad (2s)$$

where function $\varphi(B,T)$ represents the non-linear part and satisfies conditions $\varphi(B \to 0,T) \sim B$ and $\varphi(B \to \infty,T) \to 1$. We assume that the main linear contribution corresponding presumably to the Van Vleck type response of the mixed valence SmB_6 matrix is complemented by the small saturating term $\Delta M = M_{sat}\varphi(B,T)$, which may be associated with the localized magnetic moments (LMM) responsible for the ESR signal observed below characteristic temperature T^*=5.34 K.

Supplementary Note 3: Calculations schema.

In the strongly correlated electron systems, the $\varphi(B,T)$ function is not known exactly. Moreover, the microscopic nature of paramagnetic centers in the mixed valence material SmB_6 with strong charge and related spin fluctuations discovered in the present work by the ESR technique is also unknown. Therefore, it is reasonable to choose for $\varphi(B,T)$ some model form, which will allow estimating saturating magnetization M_{sat} for the LMM part ΔM of total magnetization in Equation (2s). In the present work we shall restrict ourselves with finding of this parameter only at the lowest available temperature, for which the magnitude of non-linear part of $M(B)$ is maximal, and leave the analysis of the temperature evolution of the $M(B)$ field dependences for future investigations. Thus we modeled $\varphi(B,T)$ at fixed temperature by Brillouin function $B_J(x)$ with the argument $x=\alpha(T)\cdot B$. For fixed quantum number J this function contains three free parameters (α, a and M_{sat}), which may be found from the best fit of experimental $\partial M/\partial B$ curve with the help of the derivative of Equation (2s).

Supplementary Note 4: Calculations results.

The calculation results for J=1/2 (some hypothetical paramagnetic center with the 1/2 spin) and J=5/2 (Sm^{3+} free magnetic ion) J=3/2 (a rough approximation of the Γ_8 ground state of Sm^{3+}, Ref. 40 of the main text) are shown by solid lines in Supplementary Fig. 3. The reproducibility of the experimental $\partial M/\partial B$ data is moderate and therefore this analytical expression may be used only for relatively rough estimates. However, when the parameter M_{sat} is known, it possible to find the corresponding concentration of paramagnetic centers from the standard equation $M_{sat} = Ng\mu_B J$. The sets of the parameters obtained in this way are summarized in the Supplementary Table 1; the relative concentrations of LMM were estimated for $g\approx2$ following to our ESR data (see Fig.2 in the main text).

Supplementary Note 5: Concentration estimate.

The values of N/N_{Sm} estimated from the static magnetic measurements may be treated as the upper limits for the number of localized spins responsible for the ESR in SmB_6. In any case, the spatially averaged concentration of LMM involved in dynamic magnetic response is very low and should be less than ~0.1% of total number of the Sm ions. The data obtained in the present work don't allow any definite conclusion concerning the real distribution of considered paramagnetic centers in the sample due to their unknown microscopic nature. Supposing that the

LMM are “constructed” from the Sm^{3+} magnetic ions, it is possible to expect essentially inhomogeneous distribution in space. Taking into account the experimental value of Sm valence v at T<5 K in the sample bulk v~2.52 [Supplementary reference 1] and the enhancement of this value at the sample surface up to v~2.66 as calculated in accordance with Supplementary reference 2 (see Supplementary Fig. 4) it is possible to expect that average number ~0.06% of Sm^{3+} ions may contribute to ESR, whereas at the topologically protected surface of SmB_6 this number may be enhanced by ~30%.

Supplementary References.

1. Mizumaki, M., Satoshi Tsutsui, S., and Iga, F., Temperature dependence of Sm valence in SmB_6 studied by X-ray absorption spectroscopy. *J. Phys.: Conf. Ser.,* **176,** 012034 (2009).

2. Aono, M., Nishitani, R., Oshima, C., Tanaka, T., Bannai, E., Kawai, S., LaB_6 and SmB_6 (001) surfaces studied by angle-resolved XPS, LEED, and ISS. *Surface Science*, **86**, 631 (1979).
